# The Commonwealth Caribbean COVID-19: Regions Resilient Pathway During Pandemic

**DOI:** 10.3389/fpubh.2022.844333

**Published:** 2022-05-19

**Authors:** Srikanth Umakanthan, Maryann M. Bukelo, Somu Sekhar Gajula

**Affiliations:** ^1^Department of Para-Clinical Sciences, Faculty of Medical Sciences, The University of the West Indies, St. Augustine, Trinidad and Tobago; ^2^Department of Anatomical Pathology, Eric Williams Medical Sciences Complex, North Central Regional Health Authority, Mount Hope, Trinidad and Tobago; ^3^Forensic Science Centre, St. James, Trinidad and Tobago

**Keywords:** COVID-19, caribbean, economic, regional healthcare, financial institutions

## Abstract

The coronavirus disease (COVID-19) has created severe humanitarian and socio-economic constrains in the world. The health crises caused by COVID-19 has focused on consistent co-operation and strong bonding between the developed, developing and the under-developed countries to overcome this challenging pandemic.

## Background

The Caribbean region has been exposed to numerous natural disasters and tropical diseases in the previous decade. The region formed several councils, agencies, and organizations to manage the evolving tropical infectious diseases and to maintain a stable economic platform. It was considered that the experiences gained from these calamities would motivate the Caribbean region to shield any future alarming health and financial debacles.

As COVID-19 hit the Caribbean region, the high-risk island nations had to compose adequate hospital infrastructure to tackle the roaring COVID-19 epidemic in the Caribbean. The smaller islands were prioritized to cater high standard COVID-19 care units to avoid the impetus of a devastating outcome. An increase in Intensive Care Units (ICUs) capacity would require increase in the number of fully trained hospital staff and hospital equipment's, including mechanical ventilators. Apart from health care sector, COVID-19 has hit the tourism, industry, education, and labor.

The Caribbean region has high prevalence of chronic diseases, and these diseases are the major causes of mortality and morbidity. The economically challenged population solely depend on the regional health facility for their health care and check-ups, resulting in a prolonged waiting period in the hospitals clinics. The significance of a robust health care strategy and healthcare programs should be pivotal in managing the regions epidemic.

In this commentary, we discuss the role of Commonwealth Caribbeans healthcare in combatting the ongoing COVID-19 crises, and how the region has been active in forming active healthcare and financial organizations between the regions island nations to fight the pandemic.

The COVID-19 pandemic was slow to affect the twin-Caribbean Island of Trinidad and Tobago (1). This Caribbean nation has a population of 1·395 million people and a Human Development Index (HDI) of 0.79, positioning it as the wealthiest Caribbean country. Trinidad and Tobago had reported around 130 cases of COVID-19 till July 16th, 2020. The situation was contrasting in Northern Caribbean Island, Haiti, where the COVID-19 cases surged from 85 cases in May 2020 to 8,161 cases in August 2020 (2). Haiti has an HDI of 0.51 and is positioned at 170 out of 189 countries in the World. The Haitian crisis is due to the ongoing tumultuous social and political breakdown causing a further humanitarian crisis. Other Caribbean islands (Jamaica, Aruba, Bahamas, Saint Vincent) experienced community transmission of COVID-19 during 2020 and were vigilantly monitored by the Caribbean Public Health Agency (CARPHA) by issuing frequent weekly situational reports. CARPHA was legally established in 2011 and is driven by its objective toward surveillance and management of disease by providing strategic directions to its member states (2).

Caribbean island reported its first COVID-19 case on March 10th, 2020, a lag period of almost 3 months since COVID-19 emerged in China. During this period, CARPHA, along with the Pan American Health Organization (PAHO) and the Caribbean Association of Medical Councils (CAMC), initiated swift regional epidemic control preparedness, activated incident management teams, issued updated situational reports, and statistical analysis, and developed rigid travel guidelines (2, 3). In April- May 2020, most of the Caribbean countries imposed strict border control measures and national lockdown. The control measures had a serious impact on the region's tourism sector, which forms the core of the country's economic resource in Aruba, Antigua, Bahamas, Barbados, and Dominica. COVID-19 wave caused a serious financial recession, with Aruba experiencing a GDP downfall of −13.7% in 2020. The household economic status also witnessed a serious drought as many people lost employment due to the closure of non-essential services within the Caribbean nations. Trinidad, Guyana, Suriname, and Cuba were financially surviving on their natural oil and mineral resources (4).

By June 2020, the regions health councils, in coordination with the various Caribbean national government has facilitated the individual island countries to transfer medical aids and to expand existing health care infrastructure by constructing new COVID-19 dedicated health care facility clinics, converting large public utility spaces into makeshift health care centers, increase in-hospital COVID-19 beds and ICU capacities, and medevac patients to tertiary care hospitals within the region. The CAMC, an independent non-profitable medical organization, conducted webinars to share clinical experiences within the region and receive updated COVID-19 treatment protocols from the USA and Europe, allowing access to high-standard of health care delivery within the region (5). The control measures undertaken by the Commonwealth Caribbean nations levied a heavy burden on the already stuttering region's economy. The World Bank stated that the region's economy is contracted by 7.2%, with a cumulative loss of 1.02 trillion dollars during the pandemic period of 2020–21. The region's economic crisis initiated the launch of the Caribbean Economic Recovery and Transformation plan. The economic relief provided by international financial councils (International Monetary Fund, G20's Debt Service suspension) and the perseverance of the “blue economy” has allowed the Caribbean region to sustain and safeguard its financial state during the COVID-19 pandemic era (4).

**Figure 1:**
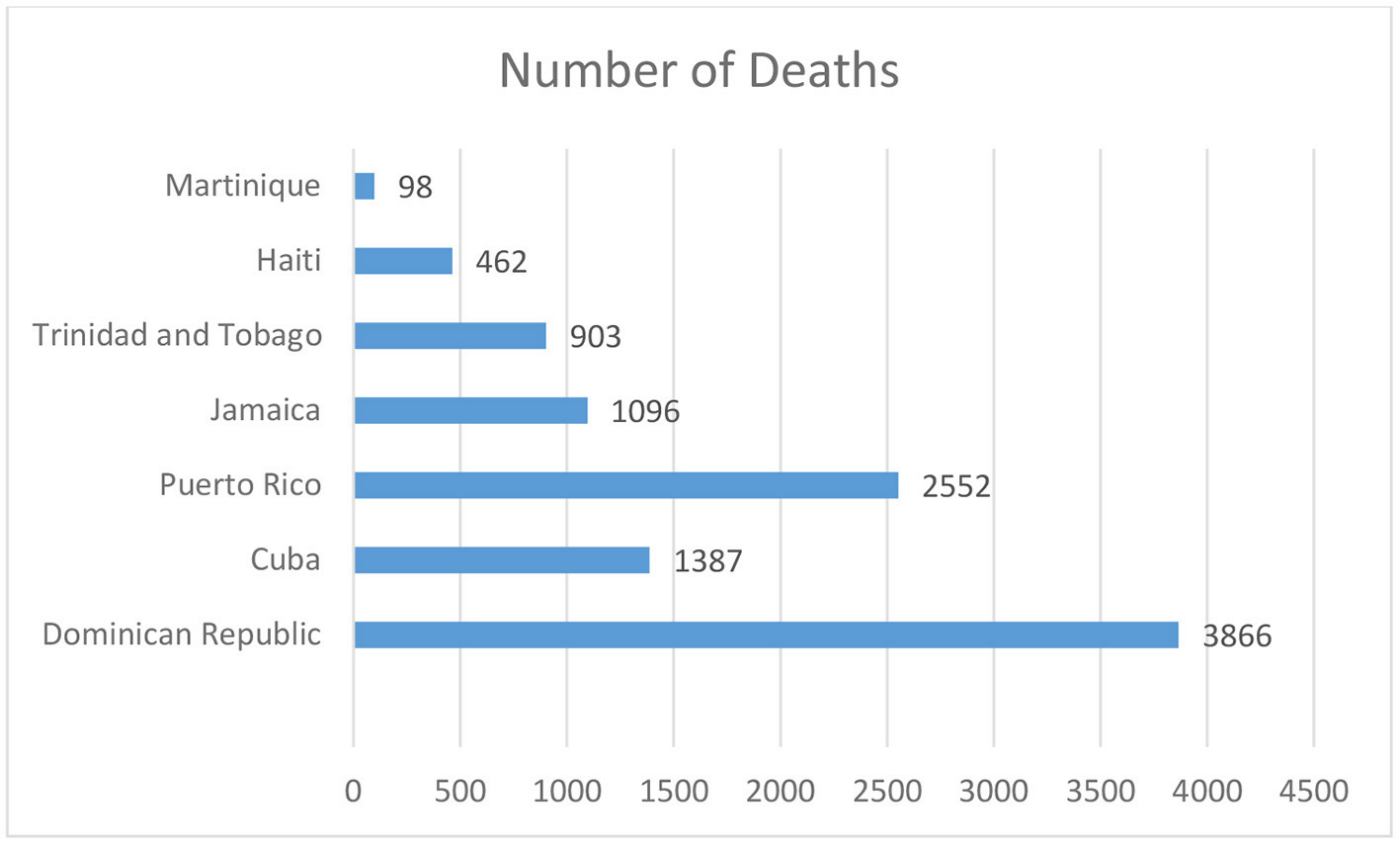
The Caribbean region COVID-19 deaths as of December 2021.

**Figure 2:**
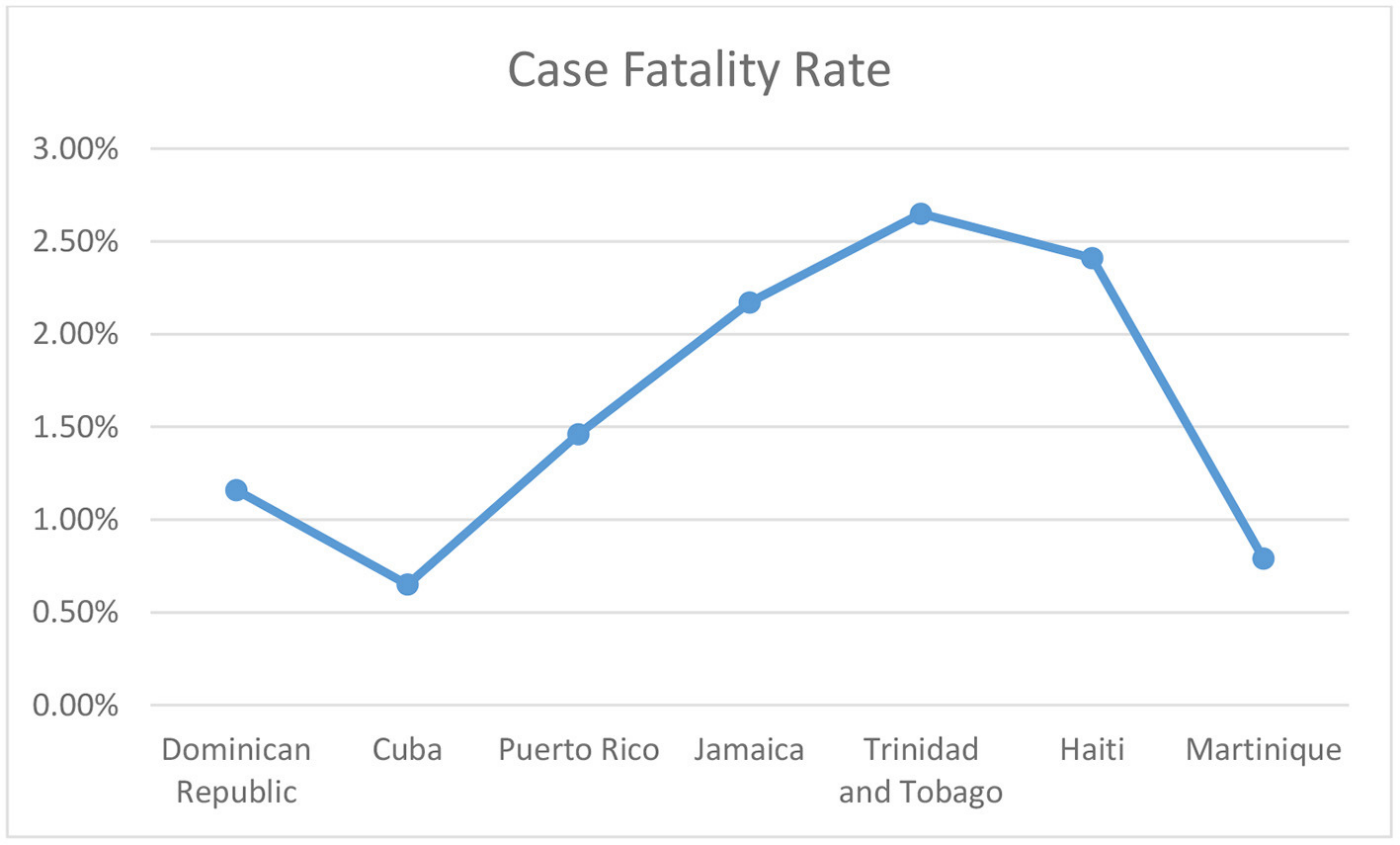
The Caribbean regions case fatality rate as of December 2021.

In 2021, the Caribbean nation's citizens experienced COVID-19 induced fatigue, which provoked the public to let their guard down, get involved in family gatherings, attend religious ceremonies, conduct election rallies, and travel around the island. The region also began to re-open its borders in a phased manner with strict regulations. The visitors were allowed into the island countries only after producing a negative RT-PCR result and a proper self-paid state quarantine to avoid further COVID-19 spikes. Jamaica created a resilient tourist corridor, providing a fort-like boundary between the locals and the tourists (6). The COVID-19 confirmed cases in the Caribbean region as of December 2021 is 2,193,737 with a case fatality rate (CFR) of 1·34%, seen in Figures 1 and 2 (2). Antigua and Barbuda is a Caribbean nation in the Lesser Antilles island chain with the countries GDP maintained by tourism, investment banking and financial-services corporations. The Bahamas constitutes 97% of the Lucayan Archipelago's land area with strong bilateral relationships with the United Kingdom and the United States of America (4, 5). The Bahamas is one of the richest countries in the Americas with its financial resilience attained by tourism, banking, agriculture, and manufacturing industries. The economy of Barbados is mixed with moderately high standards of living. The economic status of the country has waxed and waned over the years, but due to its resilient financial plan and firm trading bonds with Canada, United states of America and United Kingdom, it has been able to reduce the unemployment rate. The literacy rate in Barbados is close to 100% and the health sector is strengthened by its numerous polyclinics (6).

The role of vaccination has been the foremost global defense strategy for the fight against COVID-19. The vaccine combat against COVID-19 across the Caribbean region is initiated by exceptional collaboration by the CARPHA- Caribbean Regulatory System with the WHO and PAHO. This uniform solidarity provided timely WHO-approved vaccine supplies to the Caribbean people (7). As of the COVID-19 vaccine updated supplement provided by CARPHA, eight WHO-approved vaccines are recommended to the member states, and 14 Caribbean countries have received vaccines through the COVAX facility. The Caribbean region's average percentage of the fully vaccinated population is 29·68% (ranging from 3·8% in Jamaica to 68% in the Cayman Islands) compared to the global average of 12·7% (8). The UK has provided many AstraZeneca vaccines to its overseas territories (Anguilla, the British Virgin Islands, the Cayman Islands, Montserrat, and the Turks and Caicos Islands). The Netherlands also provides the same vaccine facility to its Caribbean counterpart (Aruba, Curaçao, and Saint Martin) (9, 10). Other nations of the Caribbean region are dependent on either bilateral deal with the vaccine-producing countries or have joined the COVAX (COVID-19 Vaccines Global Access) joint initiative. The vaccine situation remained grim in Haiti until recently, as there was no available vaccine for their citizens until COVAX delivered 500,000 doses to the nation. This inequitable severe distribution of the vaccine in the Caribbean region has often been highlighted globally and by the WHO press conferences. The pathway for successful vaccination also depended on the pivotal role of information technology within the individual islands for conducting vaccination drives and awareness programs among the public to encourage vaccination (11, 12).

## Conclusion

The Caribbean region's ability to identify community-specific requirements, recognizing self-reliance, coordinate political health care policies, and a sustainable, comprehensive health care approach has proved to be successful combat against the COVID-19 to date. Implementing rigid long-term health care and strategic financial plan using the region's COVID-19 experiences for future epidemic strikes seems to be the top priority in many Caribbean Island nations.

## Author Contributions

SU contributed in concept, design, writing the manuscript, and editing. MB and SG contributed in creating figures and data indexing. All authors contributed to the article and approved the final version.

## Conflict of Interest

The authors declare that the research was conducted in the absence of any commercial or financial relationships that could be construed as a potential conflict of interest.

## Publisher's Note

All claims expressed in this article are solely those of the authors and do not necessarily represent those of their affiliated organizations, or those of the publisher, the editors and the reviewers. Any product that may be evaluated in this article, or claim that may be made by its manufacturer, is not guaranteed or endorsed by the publisher.

## References

[1] Murphy MM Jeyaseelan SM Howitt C Greaves N Harewood H Quimby KR. COVID-19 containment in the Caribbean: the experience of small island developing states. Res Globalization. (2020) 2:100019. 10.1016/j.resglo.2020.100019

[2] Caribbean Public Health Agency (CARPHA). CARPHA Situation Reports for COVID-19. (2021). Available online at: https://carpha.org/Portals/0/Documents/COVID%20Situation%20Reports/Situation%20Report%20175%20-%20December%2015,%202021.pdf (accessed February 20, 2022).

[3] Pan American Health Organization (PAHO). COVID-19 Situation Reports. (2021). Available online at: https://www.paho.org/en/topics/coronavirus-infections/coronavirus-disease-covid-19-pandemic (accessed February 20, 2022).

[4] Hambleton IR Jeyaseelan SM Murphy MM. COVID-19 in the Caribbean small island developing states: lessons learnt from extreme weather events. Lancet Glob Health. (2020) 8:e1114–5. 10.1016/S2214-109X(20)30291-6 32622401 PMC7332258

[5] Resiere D Mehdaoui H Dyer H Chabartier C Cabié A Inamo J. Covid-19 in the Caribbean: lessons learned from the ongoing international medical and scientific cooperation. Global Health. (2021) 17:55. 10.1186/s12992-021-00706-3 33971911 PMC8107407

[6] Post L Ohiomoba RO Maras A Watts SJ Moss CB Murphy RL. Latin America and the Caribbean SARS-CoV-2 surveillance: longitudinal trend analysis. JMIR Public Health Surveill. (2021) 7:e25728. 10.2196/25728 33852413 PMC8083950

[7] Caribbean Public Health Agency (CARPHA). COVID-19 Vaccine. (2021). Available online at: https://carpha.org/Portals/0/Documents/COVID-19%20Vaccine%20Updates/CARPHA%20COVID-19%20Vaccine%20Update%20027%20December%2012,%202021.pdf (accessed February 20, 2022).

[8] Pan American Health Organization (PAHO). COVID-19 Situation: Haiti Receives 500,000 Vaccines Donated by the United States Through COVAX. (2021). Available online at: Haiti Receives 500,000 Vaccines Donated by the United States through COVAX - PAHO/WHO | Pan American Health Organization

[9] Umakanthan S Chattu VK Ranade AV Das D Basavarajegowda A Bukelo M. A rapid review of recent advances in diagnosis, treatment, and vaccination for COVID-19. AIMS Public Health. (2021) 8:137–53. 10.3934/publichealth.2021011 33575413 PMC7870385

[10] Umakanthan S Sahu P Ranade AV Bukelo MM Rao JS Abrahao-Machado LF. Origin, transmission, diagnosis and management of coronavirus disease 2019 (COVID-19). Postgrad Med J. (2020) 96:753–8. 10.1136/postgradmedj-2020-138234 32563999 PMC10016932

[11] Umakanthan S Patil S Subramaniam N Sharma R. COVID-19 Vaccine hesitancy and resistance in India explored through a population-based longitudinal survey. Vaccines. (2021) 9:1064. 10.3390/vaccines9101064 34696172 PMC8537475

[12] Umakanthan S Chauhan A Gupta MM Sahu PK Bukelo MM Chattu VK. COVID-19 pandemic containment in the Caribbean Region: a review of case-management and public health strategies. AIMS Public Health. (2021) 8:665–81. 10.3934/publichealth.2021053 34786427 PMC8568592

